# Suitability of EGCG as a Means of Stabilizing a Porcine Osteochondral Xenograft

**DOI:** 10.3390/jfb8040043

**Published:** 2017-09-23

**Authors:** Steven Elder, John Clune, Jaylyn Walker, Paul Gloth

**Affiliations:** Agricultural & Biological Engineering, Mississippi State University, Mississippi State, MS 39762, USA; jrc579@msstate.edu (J.C.); jaw1072@msstate.edu (J.W.); pdg77@msstate.edu (P.G.)

**Keywords:** osteochondral xenograft, epigallocatechin gallate, crosslinking

## Abstract

As a non-crosslinked osteochondral xenograft would be mechanically inferior to native cartilage and vulnerable to premature degradation, we seek a safe and effective method of xenograft stabilization. The purpose of this study was to evaluate the capacity for epigallocatechin gallate (EGCG) to stabilize a decellularized porcine osteochondral xenograft through collagen crosslinking. Our objectives were to assess the effects of EGCG on the degree of crosslinking, mechanical properties, collagenase resistance, cytotoxicity, and in vitro biocompatibility. EGCG is a green tea polyphenol that acts as a collagen crosslinker. Porcine osteochondral plugs were decellularized and then crosslinked by soaking in EGCG. The degree of crosslinking, cartilage compressive stiffness, cartilage-bone interface strength, coefficient of friction, and residual mass after collagenase exposure all increased with an increasing EGCG concentration. With the exception of the coefficient of friction, EGCG treatment could restore mechanical properties to levels equal to, or exceeding those, of native cartilage. EGCG treatment profoundly increased the enzymatic resistance, and 1% EGCG provided protection equivalent to 1% glutaraldehyde. EGCG up to 0.5 mM was essentially not cytotoxic to chondrocytes embedded in alginate, and autologous chondrocytes attached to decellularized, EGCG-fixed cartilage were all viable five days after seeding. Results demonstrate that EGCG has many beneficial effects on a decellularized osteochondral xenograft, and may be suitable for use in stabilizing such a graft prior to implantation for the repair of a defect.

## 1. Introduction

Focal cartilage defects in the knee are often debilitating [1], and only a few treatments immediately restore functional hyaline cartilage to the joint surface. In light of the limited availability of fresh osteochondral allografts [2] and the questionable performance of decellularized osteochondral allografts [3], it is prudent to investigate alternative approaches whereby a cartilage defect is filled with functional hyaline cartilage. The focus of the current study is a decellularized porcine osteochondral xenograft, an attractive alternative due to its widespread availability [4], potentially long storage time, and amenability to effective sterilization methods that reduce the risk of disease transmission [5]. All xenografts, including osteochondral scaffolds, must be efficiently decellularized in order to avoid adverse immune reactions [6]. Unfortunately, the trade-off for efficient decellularization of cartilage is the extraction of glycosaminoglycan (GAG) and a decrease in compressive resistance [7,8,9,10]. In addition to being mechanically inferior to normal cartilage, the decellularized xenogenic cartilage is at risk of premature enzymatic degradation in vivo [11]. Therefore, it is advisable to stabilize the xenograft through collagen crosslinking in the same manner as porcine bioprosthetic heart valves that are stabilized by using glutaraldehyde. 

Several methods of crosslinking cartilage and fibrocartilage have been investigated, including glutaraldehyde [12,13,14,15], genipin [10,13,14,15,16], proanthocyanidin [9,14], 1-ethyl-3-(3-dimethylaminopropyl) carbodiimide hydrochloride (EDC) [14,15], photo-oxidation [17], and epigallocatechin gallate (EGCG) [9,18]. For reasons of water solubility, low cytotoxicity [19,20,21], anti-inflammatory properties [19,22], cancer-fighting activity [20,23], and antibacterial effects [24], EGCG was made the subject of the current investigation. EGCG is the major catechin found in green tea (*Camellia sinensis*). It is a polyphenol that binds to collagen with a high affinity and increases its thermal and enzymatic stability [18,25]. The stabilization of collagen by EGCG is thought to involve hydrogen-bonding and hydrophobic interactions between EGCG’s galloyl moieties and side-chain functional groups on the collagen molecule [18,26,27,28]. 

EGCG has been employed to stabilize collagen membranes [29], dentin [27,30], and cartilage [9,18]. Crosslinking bovine cartilage with EGCG has been shown to increase its thermal denaturation temperature and decrease the amount of collagen and GAG released upon exposure to collagenase [18]. Direct intraarticular injections, as well as intraperitoneal injections of EGCG, have been observed to suppress the induction of collagen-induced arthritis in rats and mice, respectively [18,31]. The success of intraarticular injections was attributed to EGCG’s stabilization of collagen, such that cartilage was protected against degradation, rather than the inhibition of matrix metalloproteinases or the reduction of inflammation [18]. A previous investigation in our laboratory demonstrated that soaking in EGCG beneficially affected partially decellularized porcine cartilage [9]. Results of a ninhydrin assay indicated a substantial degree of crosslinking (reduction in the number of free amino groups), and EGCG-treated cartilage was highly resistant to degradation by collagenase. Furthermore, EGCG had no effect on the swelling ratio, and did not cause any cartilage shrinkage. Given these promising results, the current study was undertaken to examine more thoroughly the suitability of EGCG for stabilizing a decellularized osteochondral xenograft.

## 2. Results

### 2.1. Histology and SEM

**Figure 1:**
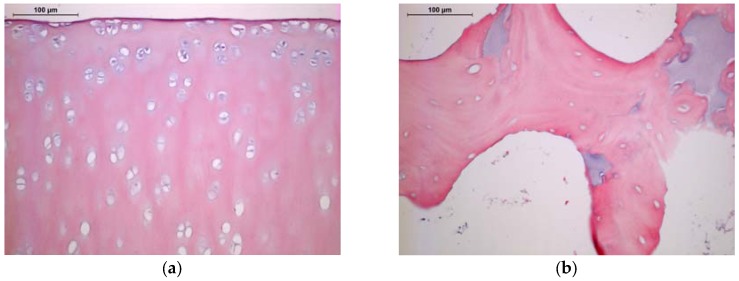
Histology of decellularized porcine stifle joint tissue. Hematoxylin and eosin staining; 200×. Scale bars = 100 μm. (**a**) Cartilage; (**b**) Bone.

**Figure 2:**
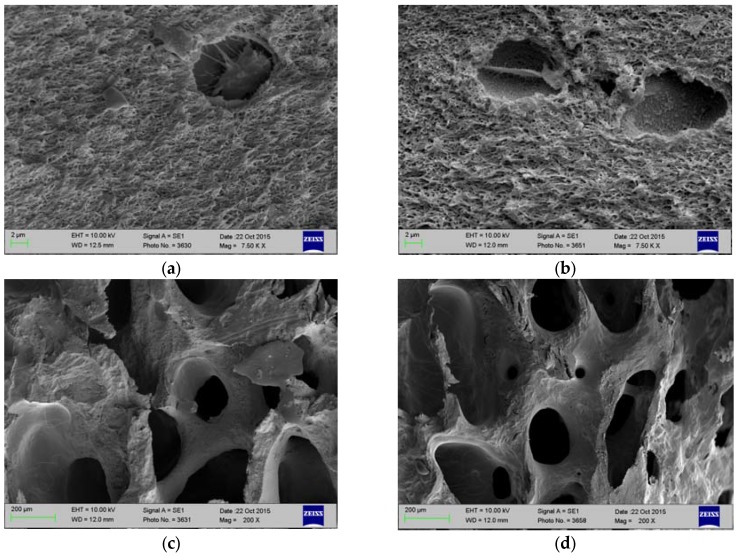
Scanning electron micrographs of cross-sections of fresh and decellularized porcine stifle joint tissue. (**a**) Fresh Cartilage; (**b**) Decellularized Cartilage; (**c**) Fresh Bone; and, (**d**) Decellularized Bone.

Efficient decellularization was confirmed by histology and SEM. Some lacunae in a zone extending approximately 50 μm from the articular surface retained nuclear remnants, but most lacunae of the decellularized cartilage were empty (Figure 1a). The bone appears to have been even more efficiently decellularized, and very few nuclear remnants were observed in osteocyte lacunae (Figure 1b). SEM also demonstrated the removal of cellular material from cartilage and bone, as well as the preservation of cartilage’s collagenous framework and bone’s trabecular morphology (Figure 2).

### 2.2. Degree of Crosslinking

**Figure 3:**
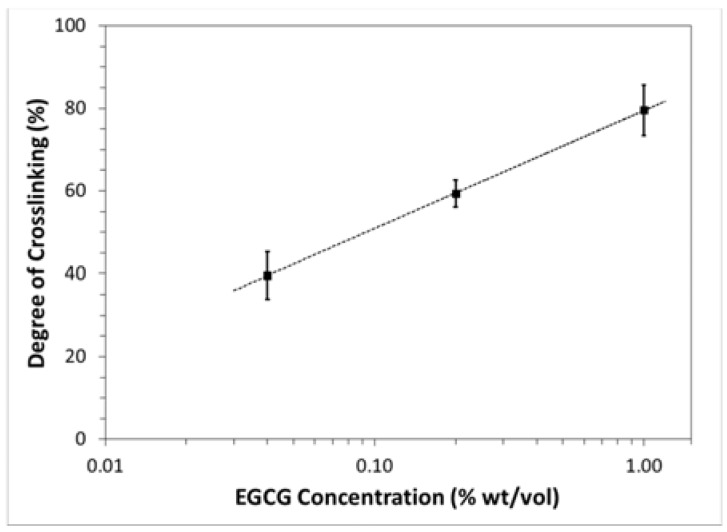
Degree of crosslinking (determined from ninhydrin assay) as a function of epigallocatechin gallate (EGCG) concentration (mean ± standard deviation). Dotted line represents result of semilogarithmic curve-fitting.

The degree of crosslinking varied with EGCG concentration in a semilogarithmic fashion (Figure 3), increasing from approximately 40% to 80% as the concentration of EGCG increased from 0.04% to 1% wt/vol. A logarithmic curve-fit of the data revealed the following relationship:

(1) Degree of crosslinking = ln[EGCG] + 12.4

where [*EGCG*] is the mass concentration of EGCG. 

### 2.3. Mechanical Properties

#### 2.3.1. Cartilage Resistance to Compression

**Figure 4:**
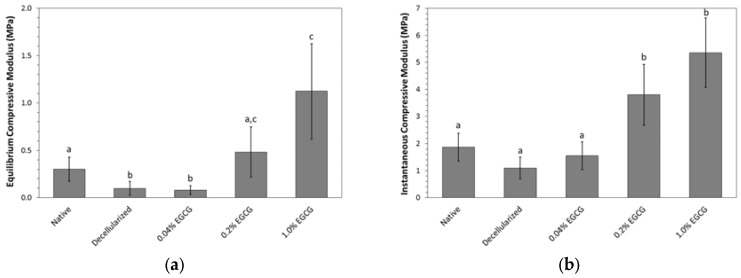
Effect of EGCG concentration on compressive resistance of decellularized porcine cartilage (mean ± standard deviation). Letters indicate statistical homogeneity as determined by ANOVA and Games-Howell post hoc test. (**a**) Equilibrium modulus; Native vs. Decellularized *p* = 0.018, Native vs. 0.04% EGCG *p* = 0.004, Native vs. 0.2% EGCG *p* = 0.448, Native vs. 1% EGCG *p* = 0.013, Decellularized vs. 0.04% EGCG *p* = 0.980, Decellularized vs. 0.2% EGCG *p* = 0.026, Decellularized vs. 1% EGCG *p* = 0.004, 0.04% EGCG vs. 0.2% EGCG *p* = 0.020, 0.04% EGCG vs. 1% EGCG *p* = 0.004, 0.2% EGCG vs. 1% EGCG *p* = 0.055; (**b**) Instantaneous modulus; Native vs. Decellularized *p* = 0.068, Native vs. 0.04% EGCG *p* = 0.635, Native vs. 0.2% EGCG *p* = 0.010, Native vs. 1% EGCG *p* = 3.75 × 10^−4^, Decellularized vs. 0.04% EGCG *p* = 0.523, Decellularized vs. 0.2% EGCG *p* = 0.001, Decellularized vs. 1% EGCG *p* = 8.27 × 10^−5^, 0.04% EGCG vs. 0.2% EGCG *p* = 0.003, 0.04% EGCG vs. 1% EGCG *p* = 1.67 × 10^−4^, and 0.2% EGCG vs. 1% EGCG *p* = 0.129.

Decellularization tended to decrease the compressive resistance of the articular cartilage as evidenced by a significant 67% reduction in equilibrium modulus (*p* = 0.018) and a 41% reduction in instantaneous modulus that trended toward significance (*p* = 0.068). EGCG crosslinking increased both moduli in a concentration-dependent manner (Figure 4). Whereas, the lowest concentration of EGCG tested, 0.04% wt/vol, did not have a significant effect on the compressive resistance of decellularized cartilage, the higher concentrations were associated with substantial and significant gains. Decellularized cartilage treated with 1% EGCG was approximately three times stiffer than the native cartilage in terms of both instantaneous and equilibrium compressive moduli.

#### 2.3.2. Cartilage-Bone Interface Shear Strength

**Figure 5:**
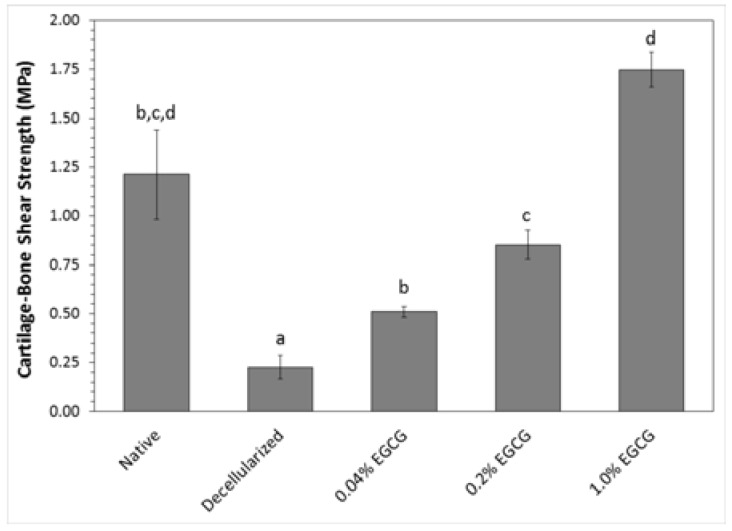
Effect of EGCG concentration on strength of the cartilage-bone interface in shear (mean ± standard deviation). Letters indicate statistically homogeneous groups determined by ANOVA and Games-Howell post hoc test; Native vs. Decellularized *p* = 0.043, Native vs. 0.04% EGCG *p* = 0.099, Native vs. 0.2% EGCG *p* = 0.310, Native vs. 1% EGCG *p* = 0.142, Decellularized vs. 0.04% EGCG *p* = 0.025, Decellularized vs. 0.2% EGCG *p* = 0.002, Decellularized vs. 1% EGCG *p* = 2.05 × 10^−4^, 0.04% EGCG vs. 0.2% EGCG *p* = 0.034, 0.04% EGCG vs. 1% EGCG *p* = 0.003, and 0.2% EGCG vs. 1% EGCG *p* = 0.001.

Results of testing the cartilage-bone interface shear strength are summarized in Figure 5. The antigen removal process drastically diminished the interface strength to approximately 20% that of native tissue. EGCG crosslinking restored the strength in proportion to its concentration. The interface shear strength of EGCG-treated samples was greater than that of the decellularized group at all concentrations tested, and there was a significant increase in strength with each increase in the EGCG concentration. 

#### 2.3.3. Cartilage Coefficient of Friction

**Figure 6:**
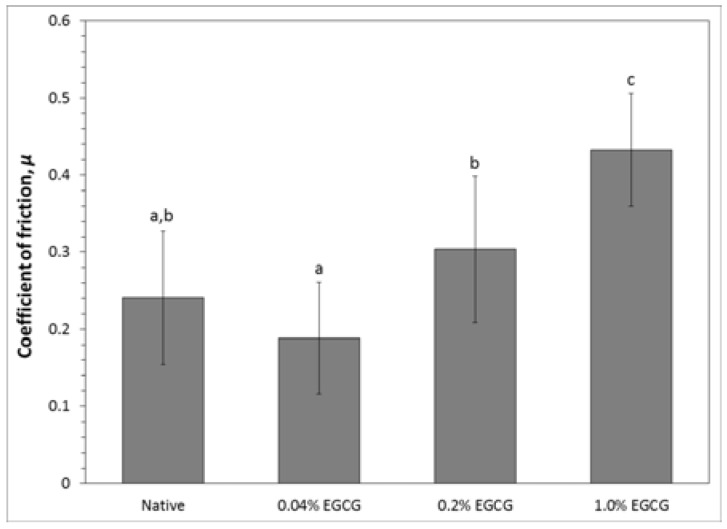
Effect of EGCG fixation on decellularized porcine cartilage coefficient of friction against glass with 20% FBS lubrication (mean ± standard deviation). The measurement of *μ* was made after 5 min of reciprocal sliding at 3.8 mm/s. Letters indicate statistically homogeneous groups determined by ANOVA and Games-Howell post hoc test. Native vs. 0.04% EGCG *p* = 0.312, Native vs. 0.2% EGCG *p* = 0.29; Native vs. 1% EGCG *p* = 1.9 × 10^−5^, 0.04% EGCG vs. 0.2% EGCG *p* = 0.016, 0.04% EGCG vs. 1% EGCG *p* = 1 × 10^−6^, and 0.2% EGCG vs. 1% EGCG *p* = 0.009.

There was a consistent trend of increasing coefficient of friction with increasing EGCG concentration. However, decellularized cartilage crosslinked with EGCG displayed a significantly higher coefficient of friction compared to native cartilage only at the highest degree of crosslinking (Figure 6). The coefficient of friction of decellularized cartilage stabilized in 1% EGCG was about 80% higher than that of native cartilage and 40% higher than decellularized cartilage crosslinked in 0.2% EGCG.

### 2.4. Resistance of Crosslinked Cartilage to Degradation by Collagenase

**Figure 7:**
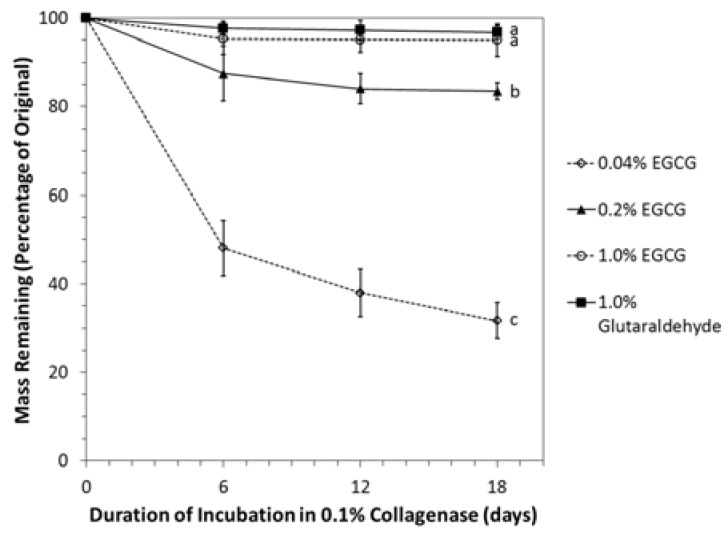
Collagenase resistance of EGCG-fixed decellularized porcine cartilage (mean ± standard deviation). Residual mass found from starting and final freeze-dried weights. Each sample was monitored over the entire 18-day evaluation period. Letters indicate statistically homogeneous groups determined by repeated measures ANOVA. Glutaraldehyde vs. 0.04% EGCG *p* = 5.9 × 10^−13^, Glutaraldehyde vs. 0.2% EGCG *p* = 6.0 × 10^−13^, Glutaraldehyde vs. 1% EGCG *p* = 0.309, 0.04% EGCG vs. 0.2% EGCG *p* = 5.9 × 10^−13^, 0.04% EGCG vs. 1% EGCG *p* = 5.9 × 10^−13^, and 0.2% EGCG vs. 1% EGCG *p* = 2.0 × 10^−10^.

The rate of mass loss upon exposure to collagenase was strongly dependent on the degree of crosslinking by EGCG (Figure 7). Non-crosslinked discs had all completely dissolved at the time of the first solution change on day 2. Discs stabilized in 0.04% EGCG lost approximately half of their mass within six days and retained only one-third of their original mass after 18 days. Discs crosslinked in 0.2% or 1.0% EGCG were more than 80% intact after 18 days, and collagenase resistance of 1% EGCG-stabilized discs was equivalent to that of discs fixed in 1.0% glutaraldehyde.

### 2.5. Cytotoxicity and Biocompatibility

**Figure 8:**
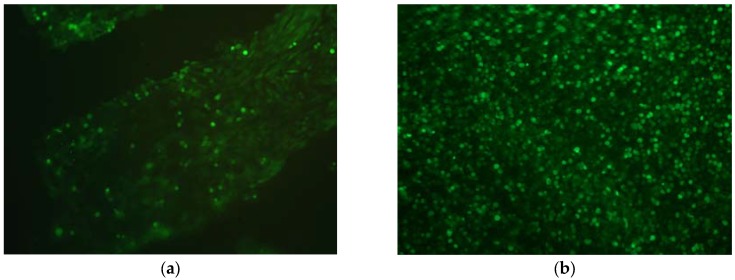
Fluorescence live/dead staining of primary porcine chondrocytes growing on decellularized porcine cartilage five days after cell seeding. Overlay of green (live cells) and red (dead cells) channels; 200×. (**a**) Cells growing on non-crosslinked cartilage; (**b**) Cells growing on cartilage treated with 1% EGCG.

The results of the cytotoxicity experiment are shown in Table 1. EGCG exhibited a very low cytotoxicity up to 0.5 mM (0.023% wt/vol). It was, however, cytotoxic at higher concentrations. The data indicate that EGCG at 5 and 50 mM killed approximately half of the cells. EGCG-fixed cartilage from which all of the unreacted EGCG had been thoroughly washed had no apparent effect on cell attachment or survival. Triplicate cell-seeded discs were examined for cell viability using fluorescence live/dead staining. All of the chondrocytes that were attached to the decellularized, EGCG-fixed cartilage discs five days after cell seeding appeared to be alive (Figure 8b). No positive red staining of nuclei (dead cells) was observed. This response was qualitatively similar to that of cells growing on non-crosslinked cartilage (Figure 8a). The only difference was that the area of cell attachment tended to be higher on EGCG-fixed cartilage than on controls. 

## 3. Discussion

Decellularization is necessary to avoid an adverse immune reaction to a xenograft, but efficient decellularization frequently compromises the tissue’s mechanical properties [32,33,34]. Crosslinking is a common approach to mechanical reinforcement of decellularized extracellular matrix scaffolds, and it may reduce antigenicity by masking the reactive groups (e.g., amino) on collagen [35,36,37]. An ideal crosslinking strategy should be simple to execute, non-cytotoxic, inexpensive, and result in a scaffold that is completely biocompatible. Glutaraldehyde is the classic crosslinking agent that has been used to stabilize xenograft heart valves for decades. However, it is extremely cytotoxic, and glutaldehyde-fixed valves are prone to calcification [38]. Alternatives to glutaraldehyde include photooxidation, carbodiimide, genipin, and plant derived polyphenols. Promising results with photooxidized osteochondral xenografts in sheep serve as proof-of-concept that a crosslinked xenograft is a potential alternative to allografts. Twelve months after surgery to repair a surgically created defect, joints carrying photooxidized xenografts exhibited a satisfactory congruency, remodeling of the subchondral bone, and cellular infiltration of the photooxidized matrix [11,17]. The disadvantage of photooxidation is the technical challenge of implementation and scaling up, as the process requires exposure to a halogen light source and constant cooling and circulation of the bathing solution. 

Carbodiimide creates amide bonds between carboxyl groups and primary amines, but it is excluded from the final crosslink. Therefore it is a zero-length crosslinker. Byproducts of the crosslinking reaction are readily washed away, and the resulting stabilized scaffold is presumably very biocompatible. However, carbodiimide-fixed decellularized porcine esophageal scaffolds implanted subcutaneously in rats did not perform as well as those crosslinked by genipin [39]. Genipin is an aglycone derived from geniposide, a naturally occurring compound in the fruit of *Gardenia jasminoides*. A growing body of research demonstrates that tissues fixed in genipin are well tolerated in vivo [40,41], and genipin certainly has beneficial effects on cartilage, which suggest its suitability for stabilizing an osteochondral xenograft [10,13,16]. We have compared genipin to plant derived polyphenols in our research and have yet to discover a clear and substantial benefit of one over the other [9]. Plant derived polyphenols include hesperidin from citrus fruits, proanthocyanidin from grape seeds, and EGCG. While we have not found serious drawbacks to any of the polyphenols, our attention was drawn to EGCG by a report of its chondroprotective effects following intraarticular injection [18]. Thus, the current study was undertaken to evaluate EGCG as stabilizer of a decellularized osteochondral xenograft.

The porcine osteochondral plugs used in this study were processed for antigen removal by a simple process that relies mostly on treatment with detergent and nucleases. Histology and SEM indicate efficient decellularization without significant disruption of the collagenous framework. We have previously reported that a very similar antigen removal protocol extracted the majority of DNA and roughly half of the GAG [10]. This alteration of the ECM can be expected to decrease cartilage compressive resistance due to a higher porosity and water permeability and lower fixed negative charge density. Furthermore, such a decellularized scaffold, without further processing, would potentially degrade faster than it could be replaced by regenerated tissue. Crosslinking the scaffold is an effective strategy for slowing rate of degradation in vivo [42,43]. 

In this study, the degree of crosslinking was proportional to the logarithm of the EGCG concentration, with 1% EGCG resulting in approximately an 80% degree of crosslinking. In a previous study, we measured the degree of cartilage crosslinking to be 52% after soaking in 0.25% EGCG. The somewhat lower degree of crosslinking than what the current study would predict could be attributable to a much shorter exposure to SDS. Retained GAG could have slowed transport of EGCG through the tissue and perhaps have inhibited the interaction between EGCG and collagen. For efficiently decellularized cartilage, the results herein suggest that soaking in 5% EGCG for 24 h at 37 °C should maximize the degree of crosslinking. For comparison, collagen membranes soaked in 0.64% EGCG for 1 h at room temperature were found to be 27% crosslinked [29]. That study demonstrated that wettability, tensile modulus, tensile strength, and thermal stability increased with an increasing EGCG concentration, while the expression of inflammatory factors decreased with an increasing degree of crosslinking. The optimal degree of crosslinking cannot be determined a priori, and it will almost certainly depend on the type of tissue and whether it has a mechanical function. For an osteochondral xenograft, a higher degree of crosslinking may be beneficial in terms of endowing the desired stiffness, suppressing inflammation, and protecting against premature degradation. On the other hand, it may impede cellular infiltration and the integration between graft and host cartilage, and it may slow the rate of degradation to the point that it inhibits new tissue formation. 

As expected, the compressive resistance of decellularized cartilage increased after treatment with EGCG in a concentration dependent manner. The gains in equilibrium and instantaneous moduli suggest that EGCG not only stiffened the solid matrix, but also lowered the hydraulic permeability. Genipin has been reported to have similar effects [10], while glutaraldehyde at concentrations up to 0.6% has been shown to increase Young’s modulus of non-decellularized bovine cartilage without significantly affecting its permeability [12]. EGCG treatment also reinforced the cartilage-bone interface, the strength of which was drastically reduced by decellularization. Cartilage-bone interface strength was restored at EGCG concentrations of 0.2% and higher. Follow-up studies in our laboratory suggest that damage to this interface can be mitigated by reducing the concentration and duration of hydrogen peroxide during the cleaning phase of the antigen removal protocol (data not shown). Decellularization and crosslinking had significant but less profound effects on cartilage coefficient of friction. The coefficient of friction of decellularized cartilage increased with increasing EGCG concentration, but only at 1% wt/vol did it become significantly greater than that of native tissue. The magnitude of the effect was approximately the same as we previously observed for decellularized cartilage crosslinked in 0.1% genipin [10]. With the substantial depletion of GAG and the loss of interstitial fluid load support after decellularization, the effect of crosslinking on frictional properties is unlikely related to alteration of the tissue permeability. Rather, it is probably a direct consequence of stiffening or roughening of collagen. Immediately after treatment with 0.2% and 0.6% glutaraldehyde, bovine calf cartilage has also been found to have a coefficient of friction roughly double that of control cartilage [12]. Interestingly, the frictional properties returned towards the control values upon incubation in phosphate buffered saline PBS and were not significantly different after 14 and 28 days. Whether such normalization occurs in vivo after EGCG crosslinking remains to be determined. 

Consistent with other studies [30,44], treating a collagenous scaffold (in this case decellularized cartilage) with EGCG drastically increased its resistance to degradation by collagenase. Although treating dissolved type I atelocollagen with just 0.1 mM EGCG (0.0046% wt/vol) has been found to provide 90% resistance to degradation by collagenase [44], we observed steady and significant increases in resistance up to 1% EGCG. Because treatment with 1% EGCG provided resistance equivalent to that from 1% glutaraldehye, we speculate that this is close to the maximum protection possible with EGCG. It has been suggested that EGCG adds structural stability to the collagen triple helix at the site vulnerable to cleavage by collagenase, thereby interfering with collagenase recognition [44]. Crosslinking with a variety of agents improves durability in vivo [39,41,45], and results of this study suggest that EGCG is no exception. 

The ideal crosslinking agent will create a totally biocompatible scaffold and will not be harmful to host cells if it leaches into adjacent tissue. This study included in vitro assessments of biocompatibility and cytotoxicity. Although some cytotoxicity was observed, it was only significant at concentrations exceeding 0.5 mM (0.023%). Nonetheless, it suggests that care should be taken to wash out EGCG from the scaffold after fixation as residual EGCG leaking from the graft could be cytotoxic to surrounding cells. There was no indication that EGCG fixation interfered with the ability of chondrocytes to attach to decellularized cartilage and survive for up to 5 days, although it is possible that some cells had died and detached before the assay was performed. Our result is consistent with a previous finding that EGCG treatment of collagen had no effect on fibroblast adhesion [44]. 

We conclude that soaking decellularized osteochondral plugs in EGCG is an effective means of crosslinking the collagenous structure, and the degree of crosslinking is readily controlled by adjusting the EGCG concentration. EGCG fixation represents a reasonable approach to restoring compressive resistance to cartilage, strengthening the bone-cartilage interface, and profoundly increasing the resistance to degradation by collagenase. Increased cartilage coefficient of friction after EGCG crosslinking is disadvantageous but is not considered a significant hindrance to use for stabilizing a xenograft. EGCG exhibits low cytotoxicity to chondrocytes, and EGCG-treated cartilage appears to be highly biocompatible. This study demonstrates that treatment of decellularized porcine osteochondral tissue with EGCG has several potential advantages, and no serious drawbacks, with respect to stabilization of an osteochondral xenograft for articular cartilage repair.

## 4. Materials and Methods 

### 4.1. Decellularization and Crosslinking

All reagents were from Sigma-Aldrich Corporation (St. Louis, MO, USA) unless specified otherwise. Stifle joints of typical market weight pigs were acquired from a local meat processor. Osteochondral plugs of 5 mm diameter × ~12 mm length were obtained from the femoral condyles of these joints using a trephine drill (A. Titan Instruments, Orchard Park, NY, USA). Antigen removal processing was carried out in 50 mL centrifuge tubes, which contained approximately 2 mL of solution per plug. All steps were carried out under rocking agitation inside a tissue culture incubator that maintained a temperature of 37 °C. The samples were first cleaned overnight in 5% hydrogen peroxide, followed by washing in phosphate buffered saline (PBS) for 60 min before further treatment. The washed samples were decellularized in a solution of 2% sodium dodecyl sulfate, 5 mM MgCl_2_, 0.5 mM CaCl_2_, 0.5 mg/mL DNase type I, 0.05 mg/mL RNase, and 1% antibiotic-antimycotic mixture for 48 h. This solution was changed after 24 h. Decellularized samples were washed twice in PBS for 60 min, and uncrosslinked controls were frozen individually in PBS at −20 °C until further use.

EGCG was dissolved in PBS at concentrations of 0.04%, 0.2%, and 1% wt/vol. Crosslinking was performed by incubating samples in these solutions for 24 h at 37 °C under rocking agitation. In order to remove residual unreacted EGCG, crosslinked samples were washed in distilled water for 1 h, changing the water every 15 min. Crosslinked samples were stored individually at −20 °C in PBS. For tests performed on cartilage alone, discs were obtained by slicing cartilage off of the bone using a scalpel, taking care to cut as close to the bone as possible.

### 4.2. Characterization of Antigen Removal by Histology and SEM

Triplicate decellularized plugs were fixed in 10% neutral buffered formalin, decalcified, and embedded in paraffin. Sections were stained with hematoxylin and eosin to demonstrate the extent of decellularization. Additional plugs were placed in Karnovsky fixative, washed in PBS, and dehydrated through graded alcohols. They were then diametrically cryofractured and further dried using ethanol and hexamethyldisilazane. After the final air drying, the samples were imaged in a JSM-6500F Field Emission Scanning Electron Microscope (JEOL Ltd., Peabody, MA, USA)). 

### 4.3. Determination of Degree of Crosslinking by Ninhydrin Assay

A ninhydrin assay was performed to determine the percentage of free amino groups (NH_2_) remaining in the crosslinked tissue after freeze drying. Ninhydrin (2,2-dihydroxyindane-1,3-dione) starts as a yellow solution that reacts with free amino groups, producing a deep purple color known as Ruhemann’s purple. A modified version of the protocol described by Cui et al. [46] was followed to prepare the ninhydrin solution and treat the cartilage discs. A microplate spectrophotometer recorded the optical absorbance of the solutions at a wavelength of 570 nm. Absorbance data were normalized to tissue dry weight, and the degree of crosslinking was calculated using the following equation:

(2) $$Degree\;of\;Crosslinking\;=\;\frac{N_{0}\;-N_{t}}{N_{0}}\;\times \;100$$

where *N*_0_ is the mole fraction of free amino groups of the control samples and *N_t_* is the mole fraction of the remaining free amino groups of the crosslinked samples.

### 4.4. Evaluation of Mechanical Properties

#### 4.4.1. Unconfined Compression of Cartilage

A Mach-1 micromechanical testing system (Biomomentum Inc., Laval, QC, Canada) was used to determine compressive resistance of cartilage discs after separation from the bone using a scalpel. Discs (*n* = 8 per group) were equilibrated to room temperature in PBS and compressed between smooth, impermeable platens. The upper platen was lowered onto the disc surface at a rate of 0.01 mm s^−1^ until the force reached 0.25 N. Disc thickness was determined from the actuator’s position relative to the bottom platen. Following the determination of thickness, stress relaxation testing was initiated by applying a strain of 5% at 0.1 mm s^−1^ and holding at that position for 180 seconds. This step was repeated four times until 25% stain was reached. Instantaneous and equilibrium moduli were calculated as the slopes of the best fit trendlines of the peak stress vs. strain and equilibrium stress vs. strain curves, respectively.

#### 4.4.2. Cartilage-Bone Interface Shear

**Figure 9:**
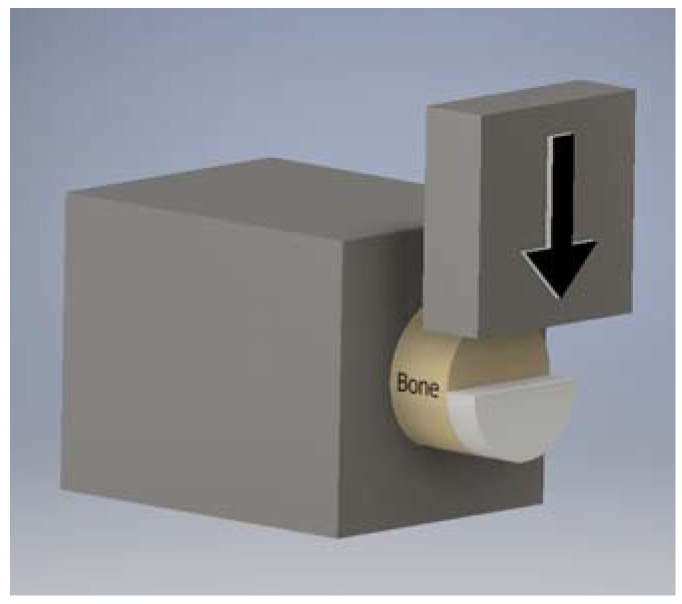
Schematic diagram of bone-cartilage interface shear testing setup.

An MTI-2K universal testing machine (Measurements Technology, Marietta, GA, USA) was used to measure the cartilage-bone interface strength of native, decellularized, and crosslinked osteochondral plugs (*n* = 3 per group). Plugs were grouted in polymethyl-methacrylate (PMMA) inside microcentrifuge tubes such that the cartilage and a short length of bone remained exposed. Using a scalpel, half of the cartilage was removed to create a shelf, and the grouted construct was secured tightly in an aluminum block such that the cartilage shelf was perpendicular to the axis of the testing machine (Figure 9). A broad, flat ram slid against the subchondral bone and sheared the remaining cartilage off by pushing against the shelf at a constant rate of 0.1 mm s^−1^. The shear strength was calculated as the maximum force divided by the semicircular area of cartilage-bone attachment.

#### 4.4.3. Cartilage Coefficient of Friction

The Mach-1 testing machine was reconfigured as previously described in order to determine the coefficient of friction of native and treated cartilage discs with a 5 mm diameter [1]. The cartilage discs (*n* = 5 per group) were affixed to the pedestal of a 105 g aluminum weight with a minuscule amount of cyanoacrylate glue, such that the weight exerted a constant normal force on the tissue. The tissue rested on a glass slide lubricated with 20% fetal bovine serum in PBS. The stepper motor platform supporting the slide underwent ramp oscillation of 19 mm amplitude at 0.1 Hz for 5 min. The coefficient of friction (*µ*) was calculated as the average frictional force (load cell) divided by the normal force during the middle third of travel in each direction. The *μ* for each sample was taken as the average over the final 3 cycles.

### 4.5. Resistance to Degradation by Collagenase

Articular cartilage discs (*n* = 8 per group) treated as stated above plus glutaraldehyde crosslinked positive controls (*n* = 8) were used for the collagenase resistance test. Glutaraldehyde crosslinking was carried out in the same way as EGCG crosslinking by incubating tissue in 2.5% glutaraldehyde prepared by diluting a 25% stock solution with PBS. Discs were freeze dried, weighed, and incubated at 37 °C under rocking agitation in 1 mL of 0.1% wt/vol type 2 collagenase (300 U/mg) in PBS containing 1% antibiotic–antimycotic mixture. The collagenase solution was replaced with fresh solution every 48 h. After six days of incubation the discs were washed twice in distilled water for 30 min and freeze dried before weighing. Discs were then returned to fresh collagenase solution for further digestion. This process was repeated after 12 and 18 days of incubation in collagenase. The percentage of mass retained was determined by dividing the dry weight at each time point by the original dry weight. 

### 4.6. In Vitro Cytotoxicity and Biocompatibility

In the event that EGCG is not completely removed after crosslinking, cells could be exposed to residual EGCG leaking from the graft. Therefore, we assessed the cytotoxicity of EGCG to articular chondrocytes. Cartilage from the distal femur of a neonatal pig was digested overnight in 0.1% wt/vol collagenase type 2, 5% fetal bovine serum, and 1% antibiotic-antimycotic solution. Released cells were suspended in DMEM/10% FBS/1% antibiotic-antimycotic mixture and seeded into polystyrene flasks at 5000 cells/cm^2^. Cells were passaged using trypsin when they were approximately 90% confluent, and three dimensional gel cultures were created using cells at the 6th passage. A suspension of 10^7^ cells/mL in 2% wt/vol low viscosity alginate was added dropwise (19 μL) to a solution containing 100 mM CaCl_2_ in 0.9% NaCl. Solidified beads were washed in DMEM and incubated in defined chondrogenic medium with TGF-β3 [47]. After five days of culture, beads were transferred to a 96-well plate, one bead per well, and exposed for one hour to EGCG dissolved in DMEM at 0.005, 0.05, 0.5, 5, and 50 mM. Cytotoxicity was evaluated based on the release of lactate dehydrogenase from dead cells as measured using a LDH Cytotoxicity Assay Kit (Cayman Chemical, Ann Arbor, MI, USA). 

Methods for testing the biocompatibility of EGCG-fixed cartilage discs to primary porcine articular chondrocytes were the same as previously published for genipin-fixed cartilage [10]. Decellularized discs were crosslinked in 1% EGCG and seeded with primary porcine articular chondrocytes. Decellularized, non-crosslinked discs served as controls. On the fifth day after cell seeding chondrocyte viability was assessed by using the Promokine Live/Dead Cell Staining Kit (PromoCell GmbH, Heidelberg, Germany). Images were captured on a Leica DM2500 epi-fluorescence microscope (Leica Microsystems Inc., Buffalo Grove, IL, USA) with Leica DFC 420C camera (Leica Microsystems Inc.) using both green and red filters to detect live and dead cells, respectively.

## Figures and Tables

**Table 1:** Cytotoxicity of EGCG based on one hour exposure to primary porcine chondrocytes embedded in alginate. Cytotoxicity was quantified by measuring amount of lactate dehydrogenase released.

EGCG Concentration (mM)	Percent Cytotoxicity
0.005	0.9
0.05	2.0
0.5	10.9
5	51.6
50	54.2

## References

[1] Heir S., Nerhus T.K., Røtterud J.H., Løken S., Ekeland A., Engebretsen L., Arøen A. (2010). A comparison of knee injury and osteoarthritis outcome score in 4 patient categories scheduled for knee surgery. Am. J. Sports Med..

[2] Nuelle C., Nuelle J., Cook J., Stannard J. (2017). Patient factors, donor age, and graft storage duration affect osteochondral allograft outcomes in knees with or without comorbidities. J. Knee Surg..

[3] Farr J., Gracitelli G., Shah N., Chang E.Y., Gomoll A. (2016). High failure rate of a decellularized osteochondral allograft for the treatment of cartilage lesions. Am. J. Sports Med..

[4] Vilsack T., Clark C. (2014). United States Summary and State Data.

[5] Pellegata A.F., Bottagisio M., Boschetti F., Ferroni M., Bortolin M., Drago L., Lovati A.B. (2017). Terminal sterilization of equine-derived decellularized tendons for clinical use. Mater. Sci. Eng. C.

[6] Keane T., Londono R., Turner N., Badylak S. (2012). Consequences of ineffective decellularization of biologic scaffolds on the host response. Biomaterials.

[7] Kheir E., Stapleton T., Shaw D., Jin Z., Fisher J., Ingham E. (2011). Development and characterization of an acellular porcine cartilage bone matrix for use in tissue engineering. J. Biomed. Mater. Res. A.

[8] Fermor H., Russell S., Williams S., Fisher J., Ingham E. (2015). Development and characterization of a decellularised bovine osteochondral biomaterial for cartilage repair. J. Mater. Sci. Mater. Med..

[9] Pinheiro A., Cooley A., Liao J., Prabhu R., Elder S. (2016). Comparison of natural crosslinking agents for stabilization of xenogenic articular cartilage. J. Orthop. Res..

[10] Elder S., Pinheiro A., Young C., Smith P., Wright E. (2017). Evaluation of genipin for stabilization of decellularized porcine cartilage. J. Orthop. Res..

[11] Akens M., von Rechenberg B., Bittmann P., Nadler D., Zlinsky K., Auer J. (2001). Long term in-vivo studies of a photo-oxidized bovine osteochondral transplant in sheep. BMC Musculoskelet. Disord..

[12] Oungoulian S.R., Hehir K.E., Zhu K., Willis C.E., Marinescu A.G., Merali N., Ahmad C.S., Hung C.T., Ateshian G.A. (2014). Effect of glutaraldehyde fixation on the frictional response of immature bovine articular cartilage explants. J. Biomech..

[13] Elder B., Mohan A., Athanasiou K. (2011). Beneficial effects of exogenous crosslinking agents on self-assembled tissue engineered cartilage construct biomechanical properties. J. Mech. Med. Biol..

[14] Zhu K., Slusarewicz P., Hedman T. (2011). Thermal analysis reveals differential effects of various crosslinkers on bovine annulus fibrosis. J. Orthop. Res..

[15] Englert C., Blunk T., Müller R., von Glasser S.S., Baumer J., Fierlbeck J., Heid I.M., Nerlich M., Hammer J. (2007). Bonding of articular cartilage using a combination of biochemical degradation and surface cross-linking. Arthritis Res. Ther..

[16] McGann M., Bonitsky C., Jackson M., Ovaert T., Trippel S., Wagner D. (2015). Genipin crosslinking of cartilage enhances resistance to biochemical degradation and mechanical wear. J. Orthop. Res..

[17] Von Rechenberg B., Akens M.K., Nadler D., Bittmann P., Zlinszky K., Kästner S.B., Auer J.A. (2006). Mosaicplasty with photooxidized, mushroom shaped, bovine, osteochondral xenografts in experimental sheep. Vet. Comp. Orthop. Traumatol..

[18] Natarajan V., Madhan B., Tiku M. (2015). Intra-articular injections of polyphenols protect articular cartilage from inflammation-induced degradation: Suggesting a potential role in cartilage therapeutics. PLoS ONE.

[19] Morin M., Grenier D. (2017). Regulation of matrix metalloproteinase secretion by green tea catechins in a three-dimensional co-culture model of macrophages and gingival fibroblasts. Arch. Oral Biol..

[20] Zeng L., Yan J., Luo L., Ma M., Zhu H. (2017). Preparation and characterization of (−)-epigallocatechin-3-gallate (EGCG)-loaded nanoparticles and their inhibitory effects on human breast cancer MCF-7 cells. Sci. Rep..

[21] Singh R., Ahmed S., Islam N., Goldberg V., Haqqi T. (2002). Epigallocatechin-3-gallate inhibits interleukin-1beta-induced expression of nitric oxide synthase and production of nitric oxide in human chondrocytes: Suppression of nuclear factor kappaB activation by degradation of the inhibitor of nuclear factor kappa. Arthritis Rheum..

[22] Fechtner S., Singh A., Chourasia M., Ahmed S. (2017). Molecular insights into the differences in anti-inflammatory activities of green tea catechins on IL-1β signaling in rheumatoid arthritis synovial fibroblasts. Toxicol. Appl. Pharmacol..

[23] Radhakrishnan R., Kulhari H., Pooja D., Gudem S., Bhargava S., Shukla R., Sistla R. (2016). Encapsulation of biophenolic phytochemical EGCG within lipid nanoparticles enhances its stability and cytotoxicity against cancer. Chem. Phys. Lipids.

[24] Kwon Y., Kim H., Hwang Y., Rosa V., Yu M., Min K. (2017). Effects of epigallocatechin gallate, an antibacterial cross-linking agent, on proliferation and differentiation of human dental pulp cells cultured in collagen scaffolds. J. Endod..

[25] Madhan B., Krishnamoorthy G., Rao J., Nair B. (2007). Role of green tea polyphenols in the inhibition of collagenolytic activity by collagenase. Int. J. Biol. Macromol..

[26] Tang H., Covington A., Hancock R. (2003). Structure—Activity relationships in the hydrophobic interactions of polyphenols with cellulose and collagen. Biopolymers.

[27] Vidal C.M., Aguiar T.R., Phansalkar R., McAlpine J.B., Napolitano J.G., Chen S.N., Araújo L.S., Pauli G.F., Bedran-Russo A. (2014). Galloyl moieties enhance the dentin biomodification potential of plant-derived catechins. Acta Biomater..

[28] Reddy R., Kumar B., Shanmugan G., Madhan B., Mandal A. (2015). Molecular level insights on collagen-polyphenols interaction using spin-relaxation and saturation transfer difference NMR. J. Phys. Chem. B.

[29] Chu C., Deng J., Xiang L., Wu Y., Wei X., Qu Y., Man Y. (2016). Evaluation of epigallocatechin-3-gallate (EGCG) cross-linked collagen membranes and concerns on osteoblasts. Mater. Sci. Eng. C.

[30] Hiraishi N., Sono R., Sofiqul I., Yiu C., Nakamura H., Otsuki M., Takatsuka T., Tagami J. (2013). In vitro evaluation of plant-derived agents to preserve dentin collagen. Dent. Mater..

[31] Lee S.Y., Jung Y.O., Ryu J.G., Oh H.J., Son H.J., Lee S.H., Kwon J.E., Kim E.K., Park M.K., Park S.H. (2016). Epigallocatechin-3-gallate ameliorates autoimmune arthritis by reciprocal regulation of T helper-17 regulatory T cells and inhibition of osteoclastogenesis by inhibiting STAT3 signaling. J. Leukoc. Biol..

[32] Liao J., Joyce E., Sacks M. (2008). Effects of decellularization on the mechanical and structural properties of the porcine aortic valve leaflet. Biomaterials.

[33] Partington L., Mordan N.J., Mason C., Knowles J.C., Kim H.W., Lowdell M.W., Birchall M.A., Wall I.B. (2013). Biochemical changes caused by decellularization may compromise mechanical integrity of tracheal scaffolds. Acta Biomater..

[34] Singh S., Afara I., Tehrani A., Oloyede A. (2015). Effect of decellularization on the load-bearing characteristics of articular cartilage matrix. Tissue Eng. Regen. Med..

[35] Gunning G., Murphy B. (2016). The effects of decellularization and cross-linking techniques on the fatigue life and calcification of mitral valve chordae tendineae. J. Mech. Behav. Biomed. Mater..

[36] Ma B., Wang X., Wu C., Chang J. (2014). Crosslinking strategies for preparation of extracellular matrix-derived cardiovascular scaffolds. Regen. Biomater..

[37] Wang X., Ma B., Chang J. (2015). Preparation of decellularized vascular matrix by co-crosslinking of procyanidins and glutaraldehyde. Biomed. Mater. Eng..

[38] Manji R., Lee W., Cooper D. (2015). Xenograft bioprosthetic heart valves: Past, present and future. Int. J. Surg..

[39] Koch H., Graneist C., Emmrich F., Till H., Metzger R., Aupperle H., Schierle K., Sack U., Boldt A. (2012). Xenogenic esophagus scaffolds fixed with several agents: Comparative in vivo study of rejection and inflammation. J. Biomed. Biotechnol..

[40] Chang Y., Tsai C.C., Liang H.C., Sung H.W. (2001). Reconstruction of the right ventricular outflow tract with a bovine jugular vein fixed with a naturally occurring crosslinking agent (genipin) in a canine model. J. Thorac. Cardiovasc. Surg..

[41] Liang H.C., Chang Y., Hsu C.K., Lee M.H., Sung H.W. (2004). Effect of crosslinking degree of an acellular biological tissue on its tissue regeneration pattern. Biomaterials.

[42] Rothamel D., Schwarz F., Sager M., Herten M., Sculean A., Becker J. (2005). Biodegradation of differently cross-linked collagen membranes: An experimental study in the rat. Clin. Oral Implants Res..

[43] Bhrany A.D., Lien C.J., Beckstead B.L., Futran N.D., Muni N.H., Giachelli C.M., Ratner B.D. (2008). Crosslinking of an oesophagus acellular matrix tissue scaffold. J. Tissue Eng. Regen. Med..

[44] Goo H., Hwang Y., Choi Y., Cho H., Suh H. (2003). Development of collagenase-resistant collagen and its interaction with adult human dermal fibroblasts. Biomaterials.

[45] Han B., Jaurequi J., Tang B.W., Nimni M. (2003). Proanthocyanidin: A natural crosslinking reagent for stabilizing collagen matrices. J. Biomed. Mater. Res. A.

[46] Cui L., Jia J., Guo Y., Lu Y., Zhu P. (2014). Preparation and characterization of IPN hydrogels composed on chitosan and gelatin crosslinked by genipin. Carbohydr. Polym..

[47] Elder S., Cooley A., Borazjani A., Sowell B., To H., Tran S. (2009). Production of hyaline-like cartilage by bone marrow mesenchymal stem cells in a self-assembly model. Tissue Eng. Part A.

